# Dermatoses gériatriques en hospitalisation dermatologique à Bamako (Mali)

**DOI:** 10.11604/pamj.2016.25.206.10568

**Published:** 2016-11-30

**Authors:** Youssouf Fofana, Bekaye Traoré, Ousmane Faye, Adama Dicko, Siritio Berthé, Koureissi Tall, Lassine Kéita, Somita Kéita

**Affiliations:** 1 Unité de Dermatologie, Centre National d’Appui à la Lutte contre la Maladie, Bamako, Mali

**Keywords:** Dermatose, hospitalisation, gériatrie, Dermatosis, hospitalization, geriatrics

## Abstract

**Introduction:**

Les pathologies cutanées du sujet âgé sont variées et constituent une véritable préoccupation pour les médecins en termes de diagnostic, de prise en charge et de suivi. Le but de ce travail était de décrire les motifs d’hospitalisation chez les sujets âgés hospitalisés dans le service de dermatologie du centre national d’appui à la lutte contre la maladie à Bamako.

**Méthodes:**

Du 1^er^ Janvier 2010 au 31 Décembre 2014, nous avons mené une étude transversale des cas de dermatoses gériatriques hospitalisées dans le service de dermatologie du centre national d’appui à la lutte contre la maladie. Sur un total de 398 patients hospitalisés, 76 malades âgés de 60 ans et plus avaient été inclus. Les données ont été saisies sur le logiciel Epidata 3.1 et analysées à l’aide du logiciel stata 14.

**Résultats:**

La fréquence des pathologies cutanées chez les sujets âgés hospitalisés était de 19,10%. L’âge des malades variait de 60 à 95 ans avec une moyenne d’âge de 68,85 ans. Les malades se répartissaient en 29 hommes et 47 femmes soit un sex-ratio de 0,60. Les principales affections recensées étaient les dermohypodermites (44,74%), les dermatoses bulleuses auto-immunes (13,16%), les toxidermies (10,53%), les ulcères veineux (6,58%), les ulcères artériels (3,95%), les tumeurs malignes (5,27%), les plaies diabétiques (3,95%). Nous avons noté 4 décès (5,26%).

**Conclusion:**

Cette étude a montré une proportion élevée des sujets âgés de 60 ans et plus en milieu dermatologique à Bamako. Par ailleurs, elle confirme que les dermohypodermites sont les pathologies cutanées les plus fréquentes chez le sujet âgé hospitalisé au Mali.

## Abstract (en)

**Introduction:**

Skin diseases in the aging population vary and are a key concern for clinicians in terms of diagnosis, management and follow-up. This study aims to describe the epidemiological and clinical aspects of skin diseases among geriatric patients hospitalized in the Department of Dermatology at the National Center In Support Of Disease, Bamako.

**Methods:**

We conducted a cross-sectional case study of patients with geriatric dermatoses hospitalized in the Department of Dermatology at the National Center In Support Of Disease from 1 January 2010 to 31 December 2014. Out of 398 patients hospitalized, 76 patients aged 60 years and over were included in the study. Data entry was accomplished using Epidata 3.1 software, while data analysis was performed using stata 14 software.

**Results:**

The prevalence of skin diseases among hospitalized geriatric patients was 19.10%. The age of the patients varied from 60 to 95 years, with an average age of 68.85 years. We enrolled 29 men and 47 women, with a sex-ratio of 0,60. The main diseases reported were dermohypodermitis (44.74%), autoimmune bullous dermatitis (13.16%), toxidermia (10.53%), venous ulcers (6.58%), arterial ulcers (3.95%), malignant tumors (5.27%), diabetic wounds (3.95%). Four patients died (5,26%).

**Conclusion:**

This study has assessed a high prevalence of subjects aged 60 and over hospitalized in the Department of Dermatology in Bamako. Moreover, it has confirmed that dermohypodermitis are the most frequent skin diseases among geriatric patients hospitalized in Mali.

## Introduction

Les maladies de peau constituent un problème majeur de santé publique dans les pays tropicaux notamment au Mali ou elles représentent, en milieu rural, la 3^ème^ cause de consultation [1]. Le processus de vieillissement, qui affecte lentement mais inexorablement le corps humain, touche particulièrement la peau. La population gériatrique étant grandissante, il est important de bien connaitre les pathologies cutanées du sujet âgé qui sont variées et qui constituent une véritable préoccupation pour les médecins en terme de diagnostic, de prise en charge et de suivi [2]. En effet, les personnes âgées peuvent présenter une grande variété de dermatoses, dont certaines peuvent avoir des conséquences graves mettant en jeu le pronostic vital. Il existe de nombreuses données sur les consultations gériatriques en dermatologie dans le monde et en Afrique mais en ce qui concerne les hospitalisations en dermatologie gériatrique, peu d’études ont été réalisées. Il n’existe aucune donnée sur ce sujet au Mali, d’où l’idée de ce travail. Le but de ce travail était de décrire les motifs d’hospitalisation chez les sujets âgés hospitalisés dans le service de dermatologie du centre national d’appui à la lutte contre la maladie.

## Méthodes

Du 1^er^ Janvier 2010 au 31 Décembre 2014, nous avons mené une étude transversale des cas de dermatoses gériatriques hospitalisées. L’étude a été réalisée dans le service de dermatologie du Centre National d’Appui à la lutte contre la Maladie (CNAM-ex institut Marchoux). Ce service situé dans la capitale du Mali (Bamako) représente le seul centre de référence dermatologique du pays. L’enquête a consisté à recenser les malades présentant une affection de peau et qui étaient hospitalisés. Ainsi, ont été inclus dans l’étude, les malades âgés de 60 ans et plus. Les malades étaient examinés par un interne et un dermatologue.

Le diagnostic dermatologique était basé sur l’examen clinique, complété au besoin par des examens biologiques et l’histopathologie cutanée. Les données ont été recueillies sur un questionnaire à partir des dossiers médicaux qui comportaient les variables démographiques (âge, sexe, scolarité, provenance), cliniques (motif de consultation, diagnostic clinique) et biologiques (tests sanguins, histopathologie cutanée, examens bactériologiques et mycologiques). Les données ont été saisies sur le logiciel Epidata 3.1 et analysées à l’aide du logiciel stata 14.

## Résultats

Durant la période d’étude, 76 malades âgés de 60 ans et plus avaient été inclus sur un total de 398 patients hospitalisés, soit une fréquence des dermatoses gériatriques de 19,10%. L’âge de nos patients variait entre 60 à 95 ans avec une moyenne d’âge de 68,85 ans. La majorité d’entre eux (69,74%) avait un âge compris entre 60 et 70 ans Tableau 1.

**Tableau 1:** Répartition des patients selon le sexe et la tranche d’âge

Sexe	Tranche d’age				
	60-70 ans	71-80 ans	81-90 ans	91-100 ans	Total
Masculin	21	3	5	0	29
Féminin	32	10	3	2	47
Total	53	13	8	2	76

Les malades se répartissaient en 29 hommes (38,16%) et 47 femmes (61,84%) soit un sex-ratio de 0,6. Selon la provenance, 55 patients provenaient des zones rurales (72,37%) et 21 provenaient des zones urbaines (27,63%). Quant à la période d’hospitalisation, nous avons enregistrés 6 malades en 2010 (7,89%), 16 en 2011 (21,05%), 16 en 2012 (21,05%), 23 en 2013 (30,26%) et 15 en 2014 (19,74%) Tableau 2. La durée moyenne du séjour était de 78,53 jours avec des extrêmes allant de 4 à 680 jours.

**Tableau 2:** Répartition des patients selon l’année d’hospitalisation

Année d’hospitalisation	Effectif	Pourcentage
2010	6	7,89
2011	16	21,05
2012	16	21,05
2013	23	30,26
2014	15	19,74
Total	76	100

Les dermatoses infectieuses étaient les plus fréquentes (47,37%) et comprenaient les fasciites nécrosantes (32,16%) suivies de l’érysipèle (6,58%) et le mycétome (2,63%) Tableau 3. La prise d’anti-inflammatoires non stéroïdiens avait été retrouvée chez 16 patients atteints de dermo-hypodermites dont 14 cas de fasciite nécrosante soit (18,42%) et 2 cas d’érysipèle soit (2,63%). Les dermatoses bulleuses auto-immunes constituaient 13,16% des motifs d’hospitalisations et comprenaient 2,63% de pemphigus et 10,53% de pemphigoïde Tableau 3. Les toxidermies représentaient 10,53% des affections recensées et incluaient 3,95% de syndrome de Lyell, 3,95% de syndrome de Stevens Johnson et 2,63% d’érythème pigmenté fixe Tableau 3. Les médicaments en cause ou fortement suspectés étaient les sulfamides (7,89%) et les anti-inflammatoires non stéroïdiens (2,63%). Les ulcères vasculaires constituaient 14,48% des motifs d’hospitalisation, principalement les ulcères veineux (6,58%), les ulcères artériels (3,95%), les plaies diabétiques (3,95%) et 1 cas d’escarre (1,32%) Tableau 3. Les dermatoses virales retrouvées étaient en la maladie de kaposi (1,32%) et le zona (1 cas, 1,32%). Les tumeurs malignes se répartissaient en 3 cas de carcinome épidermoïde (3,95%) et 1 cas de mélanome (1,32%) Tableau 3. Les dermatoses inflammatoires notées étaient l’eczéma erythrodermique (2,63%) et le psoriasis erythrodermique (1,32%) Tableau 3. Les maladies de système représentaient 1,32% des motifs d’hospitalisation, principalement la dermatomyosite Tableau 3. La sérologie était positive chez 1 des 20 patients chez qui elle avait été réalisée. Il s’agissait d’un patient atteint de pemphigoïde bulleuse.

**Tableau 3:** Répartition des patients selon les pathologies

Pathologies	Effectifs	Pourcentage
Erysipèle	5	6,58
Fasciite nécrosante	29	38,16
Pemphigus	2	2,63
Mycétome	2	2,63
Pemphigoïde	8	10,53
Ulcère veineux	5	6,58
Carcinome épidermoïde	3	3,95
Ulcere artériel	3	3,95
Escarre	1	1,32
Syndrome de Lyell	3	3,95
Stevens Johnson	3	3,95
Dermatomyosite	1	1,32
Plaie diabétique	3	3,95
Mélanome	1	1,32
Eczéma erythrodermique	2	2,63
Psoriasis erythrodermique	1	1,32
Erythème pigmenté fixe bulleux	2	2,63
Kaposi	1	1,32
Zona	1	1,32
Total	76	100

Au cours de l’évolution, la guérison avait été observée chez 44 patients, principalement les fasciites nécrosantes (28,94%), les érysipèles (5,26%), les ulcères veineux (5,26%), le pemphigus (2,63%), le mycétome (1,32%), la pemphigoïde (2,63%), le syndrome Lyell (1,32%), le syndrome de Stevens Johnson (2,63%), la plaie diabétique (1,32%), l’eczéma erythrodermique (2,63%), l’érythème pigmenté fixe bulleux (2,63%) et le zona (1,322%).

Une rémission partielle avait été obtenue chez 15 malades, principalement la fasciite nécrosante (3,95%), le mycétome (1,32%), la pemphigoïde (7,9%), l’ulcère veineux (1,32%), le syndrome de Lyell (1,32%), le syndrome de Stevens Johnson (1,32%), la dermatomyosite (1,32%), le psoriasis erythrodermique (1,32%).

Treize malades avaient été référés vers d’autres centres hospitaliers pour des raisons de complication, constituées d’érysipèle (1,32%), de fasciite nécrosante (2,63%), d’ulcère artériel (3,95%), de carcinome spinocellulaire (3,95%), de mélanome (1,32%), de Kaposi (1,32%), d’escarre (1,32%) et de plaie diabétique (1,32%).

Quatre patients sont décédés durant la période d’étude soit un taux de mortalité de 5,26%. Les principales causes du décès étaient la fasciite nécrosante (2,63%), le syndrome de Lyell (1,32%), la plaie diabétique (1,32%).

## Discussion

Le but de ce travail était de décrire les motifs d’hospitalisation chez les sujets âgés de 60 ans et plus hospitalisés de 60 ans et plus hospitalisés dans le service de dermatologie du centre national d’appui à la lutte contre la maladie à Bamako. L’étude a été réalisée dans le plus grand centre de référence dermatologique du pays sur la cohorte de consultants de cinq années. Les diagnostics retenus étaient basés sur l’examen clinique dans 61,84% et paraclinique dans 38,16%. Notre travail apporte une contribution significative à l’épidémiologie et à l’étude clinique des pathologies cutanées des sujets âgés au niveau de l’hospitalisation. L’âge de nos patients variait de 60 à 95 ans avec une moyenne d’âge de 68,85 ans et l’écrasante majorité des demandes de soins étaient formulés par les patients âgés de 60 à 70 ans (69,74%), rappelle les résultats déjà rapportés par Koussake Kombaté et al [3]. La prédominance des fasciites nécrosantes trouvées dans notre série est classiquement rapportée dans la littérature [4–6]. Cette étude avait montré que 48,28% des malades atteints de fasciites nécrosantes avaient pris auparavant des anti-inflammatoires non stéroïdiens. Ce taux est largement inferieur à celui rapporté par d’autres auteurs [6]. L’effet aggravant des anti-inflammatoires non stéroïdiens sur les dermohypodermites non nécrosantes a déjà été suggéré [5, 7, 8]. Ils pourraient avoir une action néfaste propre ou masquer les signes inflammatoires et retarder la prescription d’antibiotiques. Cette étude montrait une proportion faible des ulcères vasculaires. Ceci rejoint les données d’une étude réalisée dans 14 établissements gériatriques du Haut-Rhin (France) [9] et va à l’encontre d’une étude tunisienne [10].

Les carcinomes épidermoïdes et les mélanomes ont été rares dans notre étude contrairement aux autres séries [11–14]. Cette différence est liée au fait que notre étude a porté sur des sujets à peau génétiquement pigmentée qui ont un phototype foncé dont la mélanine épidermique plus dense agit comme un écran face aux rayons ultraviolets [15].

La maladie de Kaposi est devenue le cancer cutané le plus fréquent actuellement en Afrique subsaharienne devant les carcinomes cutanés et le mélanome [16, 17]. La faible proportion de ce cancer cutané dans notre étude était liée à la faible prévalence de l´infection à VIH chez les sujets âgés hospitalisés. Les principales étiologies des toxidermies observées chez nos patients sont superposables à celles qui sont signalées dans la plupart des études à travers le monde : il y a une prédominance nette des sulfamides anti infectieux [18–20]. Les dermatoses bulleuses auto-immunes représentaient 7,89% et ceci ne va pas dans le sens d’une étude réalisée à Lomé (Togo) [3]. Le taux de mortalité était de 5,26%, plus élevé que celui trouvé par d’autres auteurs [21].

## Conclusion

Les pathologies cutanées du sujet âgé sont multiples et dont certaines peuvent engager le pronostic vital. Les fasciites nécrosantes ont été les pathologies les plus fréquentes. Il est donc nécessaire que les dermatologues aient une compétence en gériatrie pour une meilleure prise en charge des pathologies cutanées.

### Etat des connaissances actuelles sur le sujet

- Véritable préoccupation pour les médecins;
- Les personnes âgées peuvent présenter une grande variété de dermatoses.

### Contribution de notre étude à la connaissance

- Taux de mortalité élevée;
- Faible prévalence du VIH.
